# Simultaneous Determination of Underivatized Vitamin B1 and B6 in Whole Blood by Reversed Phase Ultra High Performance Liquid Chromatography Tandem Mass Spectrometry

**DOI:** 10.1371/journal.pone.0132018

**Published:** 2015-07-02

**Authors:** Johan Puts, Monique de Groot, Martin Haex, Bernadette Jakobs

**Affiliations:** 1 Department of Clinical Chemistry and Haematology, Elisabeth-TweeSteden Hospital, Tilburg, The Netherlands; 2 Life Science group, Agilent Technologies, Amstelveen, The Netherlands; University of the Balearic Islands, SPAIN

## Abstract

**Background:**

Vitamin B1 (thiamine-diphosphate) and B6 (pyridoxal-5’phosphate) are micronutrients. Analysis of these micronutrients is important to diagnose potential deficiency which often occurs in elderly people due to malnutrition, in severe alcoholism and in gastrointestinal compromise due to bypass surgery or disease. Existing High Performance Liquid Chromatography (HPLC) based methods include the need for derivatization and long analysis time. We developed an Ultra High Performance Liquid Chromatography Tandem Mass spectrometry (UHPLC-MS/MS) assay with internal standards for simultaneous measurement of underivatized thiamine-diphosphate and pyridoxal-5’phosphate without use of ion pairing reagent.

**Methods:**

Whole blood, deproteinized with perchloric acid, containing deuterium labelled internal standards thiamine-diphosphate(thiazole-methyl-D_3_) and pyridoxal-5’phosphate(methyl-D_3_), was analyzed by UHPLC-MS/MS. The method was validated for imprecision, linearity, recovery and limit of quantification. Alternate (quantitative) method comparisons of the new versus currently used routine HPLC methods were established with Deming regression.

**Results:**

Thiamine-diphosphate and pyridoxal-5’phosphate were measured within 2.5 minutes instrumental run time. Limits of detection were 2.8 nmol/L and 7.8 nmol/L for thiamine-diphosphate and pyridoxal-5’phosphate respectively. Limit of quantification was 9.4 nmol/L for thiamine-diphosphate and 25.9 nmol/L for pyridoxal-5’phosphate. The total imprecision ranged from 3.5–7.7% for thiamine-diphosphate (44–157 nmol/L) and 6.0–10.4% for pyridoxal-5’phosphate (30–130 nmol/L). Extraction recoveries were 101–102% ± 2.5% (thiamine-diphosphate) and 98–100% ± 5% (pyridoxal-5’phosphate). Deming regression yielded slopes of 0.926 and 0.990 in patient samples (n = 282) and national proficiency testing samples (n = 12) respectively, intercepts of +3.5 and +3 for thiamine-diphosphate (n = 282 and n = 12) and slopes of 1.04 and 0.84, intercepts of -2.9 and +20 for pyridoxal-5’phosphate (n = 376 and n = 12).

**Conclusion:**

The described UHPLC-MS/MS method allows simultaneous determination of underivatized thiamine-diphosphate and pyridoxal-5’phosphate in whole blood without intensive sample preparation.

## Introduction

Thiamine, the first B vitamin identified, exists as unphosphorylated and as phosphorylated thiamine derivatives; thiamine-monophosphate (TMP), thiamine-diphosphate (TDP) and thiamine-triphosphate (TTP) [1]. The main biological active form is TDP, which is present in erythrocytes. TDP plays an essential role as a coenzyme in the decarboxylation of α-ketoacids and branched-chain amino acids. Therefore it is essential for energy generation (Krebs-cycle). In addition, TDP acts as a coenzyme for the transketolase reaction that mediates the conversion of hexose and pentose phosphates [1]. Since thiamine is a water-soluble vitamin and body stores are limited, deficiency occurs within 2–3 weeks of cessation of intake [2]. Deficiency of thiamine is known to cause beriberi and Wernicke’s encephalopathy, which can be found in alcohol-dependent and elderly people. Moreover, it has been linked to microvascular complications in patients with Diabetes Mellitus [1,3–5]. It has been postulated that thiamine plays a role in peripheral nerve conduction, although the exact chemical reactions underlying this function are not known.

Vitamin B6 refers to a family of compounds including pyridoxine (PN), pyridoxal (PL), pyridoxamine (PM) and their 5′phosphate derivatives. Pyridoxal-5’phosphate (PLP) is the active form of vitamin B6 which acts as a cofactor for more than 140 enzymes involved in the metabolism of amino acids, glycogen, lipids, steroids and several vitamins, including the conversion of tryptophan to niacin as well as involvement in haem and neurotransmitter synthesis [6, 7]. Deficiency of PLP can occur in elderly people due to malnutrition and in gastrointestinal compromise due to bypass surgery and disease. In addition, its essential role in normal brain development and functioning has been recognized. Specific inborn errors of metabolism resulting in functional B6 deficiency have been identified [8]. On the other hand ingestion of large doses of B6 have been reported to be toxic leading to polyneuropathy [9,10]. The mechanism of this neurotoxicity has not been elucidated yet.

The most frequently used method for analysis of TDP and PLP is a HPLC-based assay for each vitamin separately [11–25]. Indeed, the currently used HPLC-based methods in our laboratory for measuring TDP and PLP consist of two separate HPLC methods. TDP is analyzed at pH 10 by reversed phase liquid chromatography performing a pre-column derivatization step with alkaline potassium ferricyanide and fluorescence detection as described by van Landeghem et al [11]. PLP is pre-column converted to a semi-carbazone followed by column separation and fluorescence detection at pH12 according to Schrijver et al [12]. These methods are labour and time consuming and precautions should be taken since cyanide and semicarbazide, even in small amounts, are toxic reagents. Furthermore, the HPLC-based assay for each vitamin is performed in about 20 minutes. Our goal was to develop for patient samples a method on UHPLC-MS/MS with simple sample preparation and no derivatization to identify TDP and PLP levels in one short run without using an ion pairing reagent such as heptafluorobutyric acid.

## Materials and Methods

### Ethics statement

Leftover patient blood samples were anonymized and re-used for validation of the method. This procedure was exempted from the Medical Research Involving Human Subjects Act (WMO) and approved by the Medical Ethics Committee as it did not meet the criteria for medical-scientific research. Patients are informed via the laboratory request form about the possibility of further test-related research or quality control (QC) on routine patient samples. Patients are given the opportunity to declare a no-cooperation statement for this procedure.

### Materials

Thiaminediphosphate (TDP) and pyridoxal-5’phosphate (PLP) were purchased from Sigma-Aldrich (St. Louis, USA).

Deuterium labelled internal standards TDP (thiazole-methyl-D_3_) and PLP (methyl-D_3_) were supplied by Cambridge Isotope Laboratories Inc. (Tewksbury, USA).

Perchloric acid (70%), hydrochloric acid and ammonium acetic acid, pro analyse were obtained from Merck (Darmstad, Germany), ammonium carbonate (>30.0% NH_3_ basis) from Sigma-Aldrich and methanol (100% gradient grade) from Merck.

All reagents were of high purity grade designated for high performance liquid chromatography and mass spectrometry.

Water was obtained from a demi water system of Ovivo, Mettler-Toledo B.V. (Zurich, Switzerland).

Leftover blood samples (n = 12) were collected from patients who presented to the hospital for laboratory analyses of TDP and PLP. Blood samples were pooled and used as matrix material for the calibration curve. The initial values of TDP and PLP were 78 nmol/L and 55 nmol/L, respectively, as established by standard addition method on the UHPLC-MS/MS. Part of the pooled whole blood sample was spiked with TDP 75 and 150 nmol/L and with PLP 50 and 100 nmol/L, aliquoted and stored at -80°C until use for recovery experiments.

### Sample collection and processing

Blood samples, collected in K_2_-EDTA vacutainer tubes (Becton Dickinson), were immediately stored at -20°C upon arrival in the laboratory. Before analysis, samples were prepared as reported previously for TDP by van Landeghem et al [11], with minor modifications: 2 ml of cold 5.25% perchloric acid (aq), containing internal standards TDP-D_3_ (114 nmol/L) and PLP-D_3_ (138 nmol/L) was added to 0.5 ml haemolysed blood plus 50 μl water. The distilled water was added to equalize the volumes of the standards and the samples. Tubes did not need to be kept at 4°C and because the LC-system is equipped with a 0.2 μM filter, right after the needle seat of the auto-sampler which avoid particles to block the column, filtration of the samples was not necessary. After vortexing, samples were centrifuged for 15 min at 3500*g* at 4°C and an aliquot (0.5 ml) was transferred into light protected vials and placed in the auto-sampler tray of the UHPLC-MS/MS system.

### Preparation of internal standards

TDP-D_3_ (3.9 mg) and PLP-D_3_ (1.4 mg) were separately dissolved in 10 ml 0.01 M HCL.

### Preparation of standards

The stock standards, prepared in 0.01 M HCL, contained 3.0 mmol/L of TDP and 0.5 mmol/L of PLP. The concentration of TDP and PLP were determined by weighing the dry chemicals. Exact concentration of TDP and PLP were determined by spectrofotometric measurements at 248 nm and 295 nm, respectively. Working standards were prepared by diluting the stock standards to 1500 nmol/L TDP and 1000 nmol/L PLP with 0.01 M HCL.

The calibration curve was composed by adding PLP and TDP to 500 μL whole blood as follows: 50 μL water: 500 μL whole blood (standard 1), 25 μL water plus 25 μL working standard: 500 μL whole blood (standard 2), 50 μL working standard: 500 μL whole blood (standard 3). Whole blood is used for the calibration curve to mimic patient samples.

### UHPLC analysis

The UHPLC system used was a 1290 Infinity Binary LC equipped with a 0.2 μM filter, right after the needle seat of the autosampler system (Agilent Technologies, Santa Clara, USA). Two columns were tested for separation of TDP and PLP: **I)** 100x 2.1 mm Hypercarb Porous Graphitic Carbon LC column 3 μM particle size (Thermo Fisher Scientific Inc., Waltham, USA) equipped with a 10x2.1 mm Hypercarb Drop-in guard cartridge 3 μM particle size and **2)** 50x2.1 mm Zorbax Eclipse Plus C18, 1.8 μm particle size equipped with a 5x2.1 mm Zorbax Eclipse plus C18, 1.8 μM particle size pre column (Agilent Technologies). Two mobile phases were tested: **1)** 50 mmol/L ammonium acetic acid (pH 6.8) and **2)** 98% water + 4.8 g/L ammonium carbonate (50 mmol/L) and 2% methanol (pH 9.5).

Two column temperatures tested were 5°C and 10°C.

Optimal separation analysis of TDP and PLP was accomplished with: The column and auto-sampler temperatures were 5°C and room temperature, respectively. The separation analysis was performed by injecting 10 μl on a 50x2.1 mm Zorbax Eclipse Plus C18, 1.8 μm particle size column equipped with a 5x2.1 mm Zorbax Eclipse plus C18, 1.8 μm particle size pre column (Agilent Technologies). The mobile phase consisted of 98% water + 4.8 g/L ammonium carbonate (50 mmol/L) and 2% methanol (pH 9.5).

The compounds of interest were isocratically eluted at a flow rate of 0.4 ml/min. The length of the analytical run was 2.5 min.

### MS detection

MS detection was performed on a 6460 MS system (Agilent Technologies) with a Jetstream interface. In order to prevent contamination of the ion source, the MS was set to waste for the first 0.45 minutes to wash out polar contaminants.

Optimized parameters for the jet stream ionization source and MS system were established with pure internal standards TDP-D_3_ and PLP-D_3_. All ions were analyzed in a positive mode.

### Intra and inter day precision of the assay

Three pools of whole blood with low, medium and high concentrations of TDP (44, 104, and 157 nmol/l) and PLP (30, 90, 130 nmol/l) were aliquoted and stored at –80°C. In one analytical run the different pools were analyzed in four fold, two samples at the beginning and two samples at the end of a run. The inter-day precision of the assay was established by measuring the pools on 10 days over a period of 2 weeks (n = 4 analysis per run *10 days = 40). A between-day variance lower than 15% (CV<15%) was set for test acceptance according to Honour et al [26].

### Linearity

To establish linearity whole blood collected from patients with initially low concentrations of TDP (112 nmol/L) and PLP (39 nmol/L) was pooled (= low, pool concentration).1075 nmol/L TDP and 2825 nmol/L PLP was added to this low pool to create the high concentration pool. The low and high sample pool were mixed in different ratios according to Clinical and Laboratory Standards Institute protocol evaluation protocol 6 (CLSI protocol EP6) [27], to create samples with 5 concentrations and analyzed by UHPLC-MS/MS. The various sample pools were also analyzed with the routine HPLC method.

### Recovery

The influence of the matrix was established as follows: 12 patient samples were prepared according to the standard procedure;12 patient samples were 1:1 diluted with H2O; 12 patient samples were spiked with TDP (75 and 150 nmol/L) and PLP (50 and 100 nmol/L). 14 patient samples were analyzed with half the injection volume. The procedure was in accordance with CLSI protocol EP 7 [27].

### Alternate (quantitative) comparison of methods

Patient samples (TDP: n = 142, concentration range 91–312 nmol/L; PLP: n = 188, concentration range 29–573 nmol/L) were analyzed in duplicate by the routine HPLC method (TDP, thiochrome method, according to van Landeghem et al [11]; PLP, semicarbazide method, as described by Schrijver et al [13]) and on the same day by the new UHPLC-MS/MS method. Moreover, samples of our national proficiency testing program and commercially available control levels of Chromsystems (Munich, Germany), Recipe (Munich, Germany) and Instruchemie (Delfzijl, The Netherlands) were analyzed with both methods.

### Sensitivity of Quantitation

Lower limits of detection and quantification (LOD, LOQ) were established using the Agilent Tandem MS software and the internal standards. LOD and LOQ were calculated by analyzing the low standard 10 times. The LOD was defined as the concentration at which the signal-to-noise (S/N) ratio was 3 and the LOQ was defined as the concentration with a S/N ratio of 10.

### Statistical analysis

Data and statistical analysis i.e. evaluation of intra/interday precision of the assay, EP6, EP7 and Alternate (Quantitative) Method Comparison (Deming regression) were performed using EP Evaluator release 7 software, Data Innovations LLC (Brussels, Belgium).

## Results

### Tuning of the mass spectrometer

By infusing pure solutions of TDP, TDP-D_3_, PLP and PLP-D_3_ (1 mg/10 ml 0.01 M HCL) into the Jetstream ionization source of the tandem MS, optimal set points for the Jetstream ionization source and MS system were determined (Table 1A). All ions were analyzed in positive mode. The ion transitions for TDP, TDP-D_3_, PLP and PLP-D_3_ at 3V acceleration voltage are shown in Table 1B. The ions were confirmed by the NIST 2014 Mass Spectral Library, version 2 (Agilent Technologies, Amstelveen, The Netherlands).

**Table 1:** Tandem Mass spectrometer (MS/MS) settings and conditions.

**A: Mass spectrometer settings**		
**Gas Temp**	**200**	**(°C)**
**Gas flow**	**10**	**(L/min)**
**Nebulizer pressure**	**40**	**(psi)**
**Sheat gas heater**	**350**	**(°C)**
**Sheat gas flow**	**11**	**(L/min)**
**Capillary voltage**	**3500**	**V**
**Charging voltage (+ ion mode)**	**500**	**V**
**B: Collision energy (CE) for different multiple-reaction monitoring transitions**		
**TDP-D**_**3**_	**428>125**	**(CE 20 ev)**
**TDP**	**425>122**	**(CE 20 ev)**
**TDP**	**425>81**	**(CE 52 ev)**
**PLP-D**_**3**_	**251>153**	**(CE 12 ev)**
**PLP**	**248>150**	**(CE 12 ev)**
**PLP**	**248>94**	**CE (28 ev)**

### Chromatography

**Fig 1:**
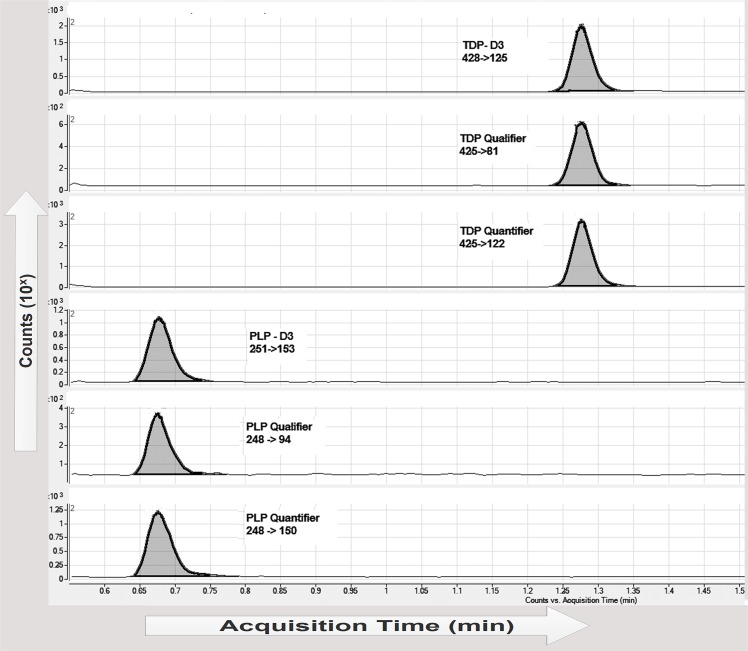
UHPLC-MS/MS elution profiles of human whole blood TDP, PLP, and internal standards in a total run time of 2.5 min. TDP is thiamin-diphosphate, PLP is pyridoxal-5’phosphate. Specific mass transitions (*m/z)* are shown in the figure.

Several columns (a 100x 2.1 mm Hypercarb Porous Graphitic Carbon LC column 3 μM particle size equipped with a 10x2.1 mm Hypercarb Drop-in guard cartridge 3 μM particle size, a 50x2.1 mm Zorbax Eclipse Plus C18, 1.8 μm particle size equipped with a 5x2.1 mm Zorbax Eclipse plus C18, 1.8 μM particle size pre column), mobile phases (50 mmol/L ammonium acetic acid (pH 6.8), 98% water + 4.8 g/L ammonium carbonate (50 mmol/L) and 2% methanol (pH 9.5)) and columns temperatures (5°C and 10°C) were performed to achieve separation of TDP and PLP in one analytical run. The hypercarb column showed a 10 bar increase of pressure after every new sample injection, which could not be lowered with any type of column cleaning. Weekly around 150 samples TDP and PLP have to be analysed, meaning that 10 bar increase of column pressure after every sample is not a robust method to use in a routine setting. The best TDP and PLP separation results were accomplished with a 50x2.1 mm Zorbax Eclipse Plus C18, 1.8 μm particle size equipped with a 5x2.1 mm Zorbax Eclipse plus C18, 1.8 μM particle size pre column at a column temperature of 5°C. The optimal pKa for TDP and PLP showed to be around pH 9.5. Using a mobile phase of ammonium carbonate (50 mmol) and methanol (2%) (pH 9.5) to isocratically elute PLP and TDP, retention times achieved were respectively 0.67 min and 1.27 min with a total analysis time of 2.5 min (Fig 1).

Although this conditions (high pH, % organic solvent and mM buffer) are on the edge of the companies advised condition of the column, testing showed that the column is robust enough to handle these conditions (> 2500 samples at pH 9.5 without replacing /changing the column).

### Minimal Detection

The LOD is 2.8 nmol/L and LOQ is 9.4 nmol/L for TDP. The LOD is 7.8 nmol/L and LOQ is 25.9 nmol/L for PLP.

### Precision/Imprecision

Imprecision of the method estimated by low, medium and high concentrations of TDP and PLP is shown in Table 2. The intra- and inter day precisions were 2.4–4.1% and 2.4–6.5% for TDP (44–157 nmol/L), respectively and 5.5–5.5% and 2.2–8.8% for PLP (30–130 nmol/L), respectively. The total imprecision ranged from 3.5–7.7% for TDP and 6.0–10.4% for PLP.

**Table 2:** Imprecision of TDP and PLP analysis in whole blood on 10 consecutive days (n = 40).

Compound	Mean	Intra day		Inter day		Total	
	nmol/L	SD	CV (%)	SD	CV (%)	SD	CV (%)
**PLP**	**30**	**1.6**	**5.5**	**2.6**	**8.8**	**3.1**	**10.4**
**PLP**	**90**	**5.3**	**5.9**	**1.7**	**1.9**	**6.4**	**7.1**
**PLP**	**130**	**7.2**	**5.5**	**2.9**	**2.2**	**7.7**	**6.0**
**TDP**	**44**	**1.8**	**4.1**	**2.9**	**6.5**	**3.4**	**7.7**
**TDP**	**104**	**3.7**	**3.6**	**1.6**	**1.6**	**4.2**	**4.0**
**TDP**	**157**	**3.7**	**2.4**	**3.8**	**2.4**	**5.5**	**3.5**

### Linearity

Linearity evaluated with 5 concentrations of TDP and PLP revealed that both components were linear. TDP was linear up to 1153 nmol/L with a slope of y = 0.985x+6.9 (r = 0.999). PLP was linear up to 2864 nmol/L with a slope of y = 1.013x-11.2 (r = 0.999).

### Recovery

Recoveries of PLP and TDP in 1:1 water: pooled whole blood were +10% for PLP and +20% for TDP. Instead of sample dilution, injecting a smaller sample volume is preferable as this showed recoveries of 100% for PLP and 101% for TDP (Table 3).

**Table 3:** Recovery of PLP and TDP added to whole blood samples and dilution matrix effect compared to less sample injection.

Sample	Number of samples	Mean %	SD %	(Individual 95% CI)
**PLP 2 x diluted**	**12**	**110.1**	**7.9**	**(5.4;13.7)**
**PLP + 50 nmol/l**	**12**	**98.5**	**4.2**	**(2.8;7.9)**
**PLP + 100 nmol/L**	**12**	**99.7**	**5.5**	**(4.1;8.8)**
**PLP ½ injection vol.**	**12**	**100.4**	**3.7**	**(2.6;6.1)**
**TDP 2 x diluted**	**12**	**120.4**	**4.4**	**(2.6;8.9)**
**TDP + 65 nmol/L**	**12**	**100.9**	**2.5**	**(2.0;3.7)**
**TDP + 150 nmol/L**	**14**	**101.9**	**2.5**	**(2.0;3.8)**
**TDP ½ injection vol.**	**14**	**101.2**	**2.3**	**(1.5;4.2)**

### Method comparison

Methods were compared over a period of a month using patient samples (n = 188 for PLP and n = 142 for TDP). Sample concentrations varied from 91 to 312 nmol/L for TDP and from 29 to 573 nmol/L for PLP as established by HPLC methods.

**Fig 2:**
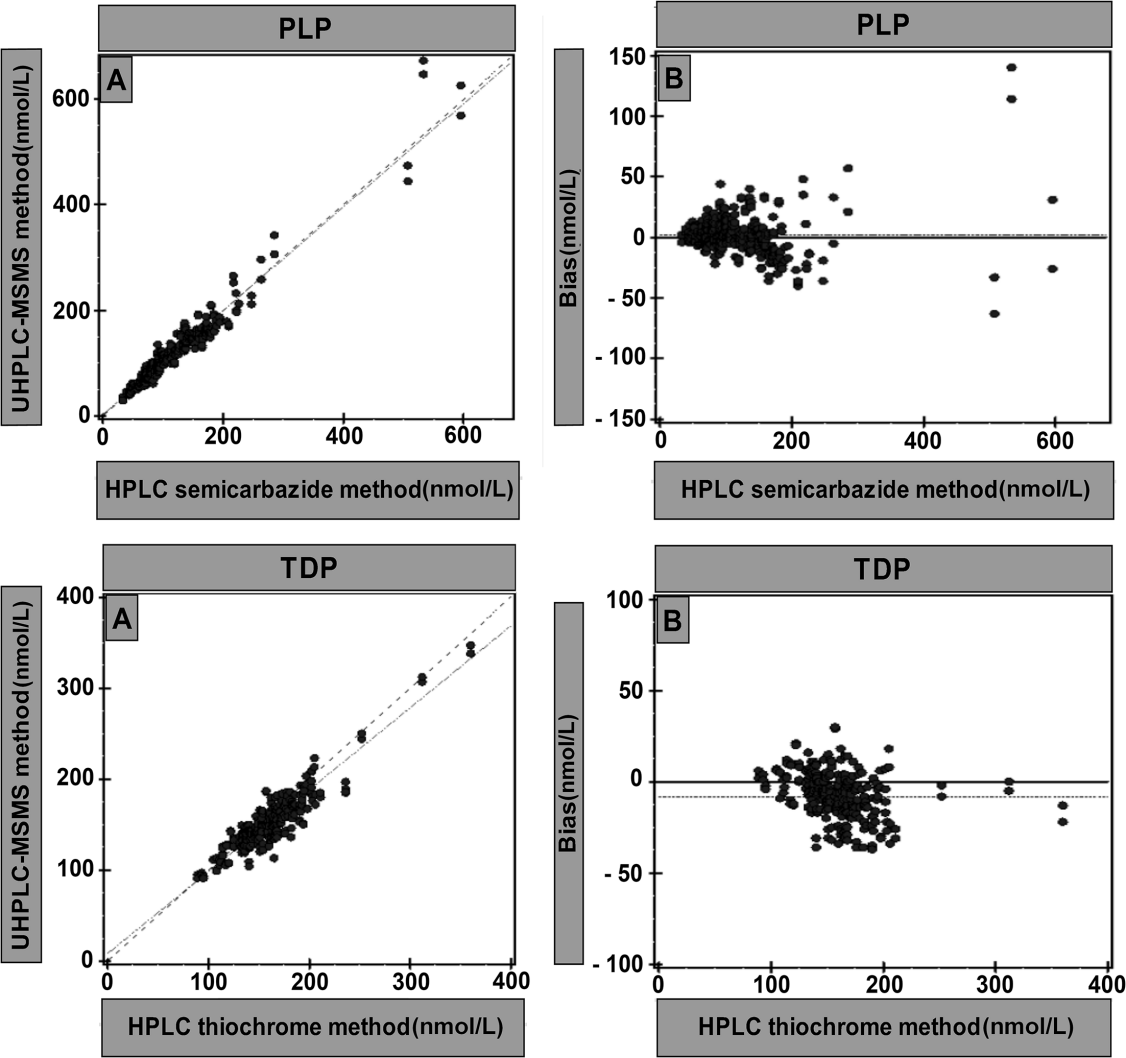
Deming regression of the HPLC methods and UHPLC-MS/MS method for estimating PLP and TDP. A = Scatter plot (nmol/L), B = Bias plot (nmol/L).

Deming regression for PLP yielded y = 1.040x -2.9 (n = 376, in duplicate) r = 0.974, average bias 2.0 (1.6%) where x = PLP semicarbazide method and y = UHPLC-MS/MS method. Deming regression for TDP yielded y = 0.926x + 3.5 (n = 282, in duplicate) r = 0.910, average bias -8.4 (-5.4%) where x = TDP thiochrome method and y = UHPLC-MS/MS method (Fig 2).

**Fig 3:**
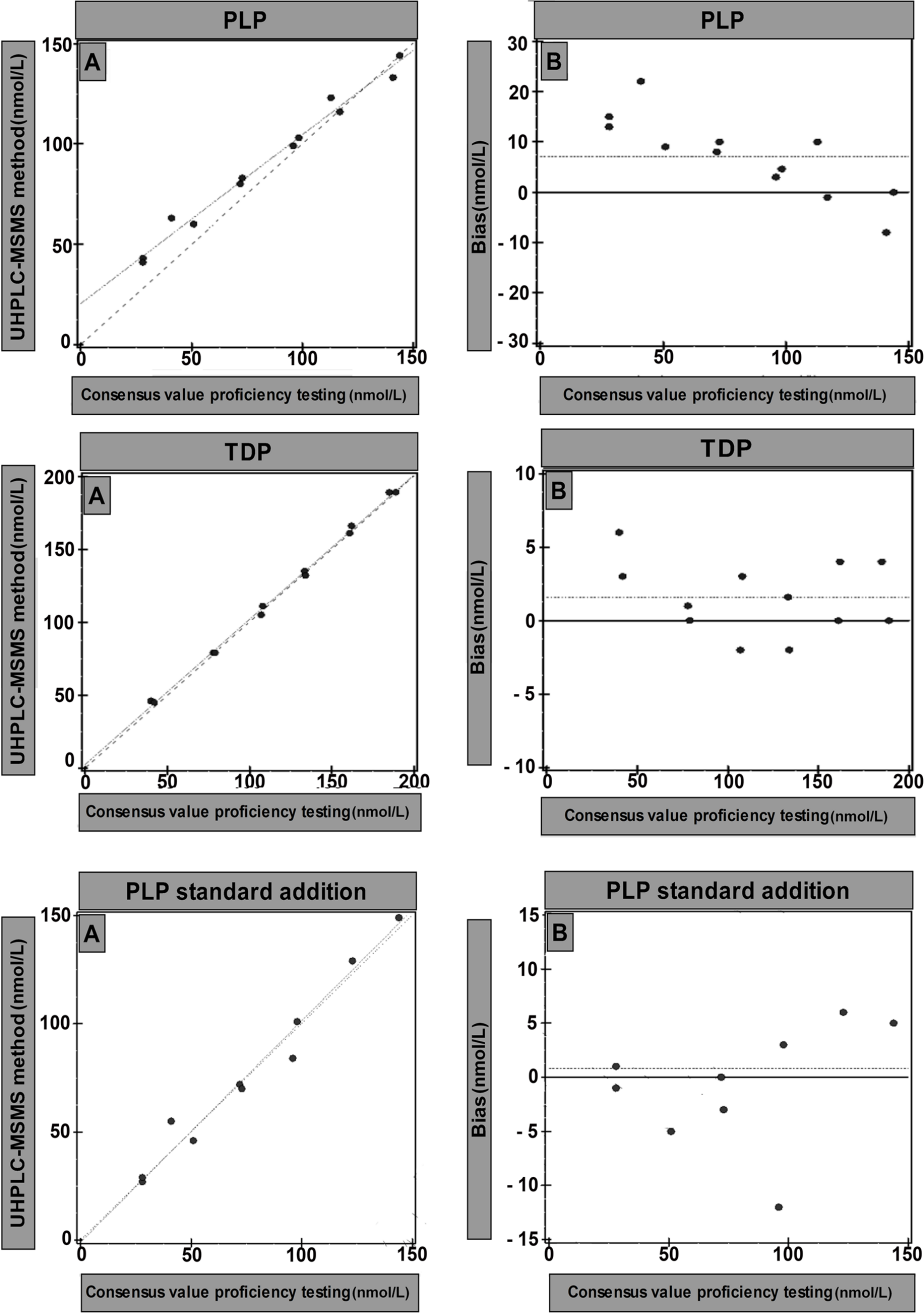
Deming regression of the UHPLC-MS/MS method versus consensus value of samples of our national proficiency testing program. A = Scatter plot (nmol/L), B = Bias plot (nmol/L).

Comparison of the UHPLC-MS/MS method with our national proficiency testing program samples (n = 12) by Deming regression yielded a slope of 0.990 with an intercept of 2.68, r = 0.998 and average bias of 1.55 (1.3%) for TDP and a slope of 0.84 with an intercept of 20.49, r = 0.992 and average bias of 7.13 (8.19%) for PLP (Fig 3).

### Stability

**Fig 4:**
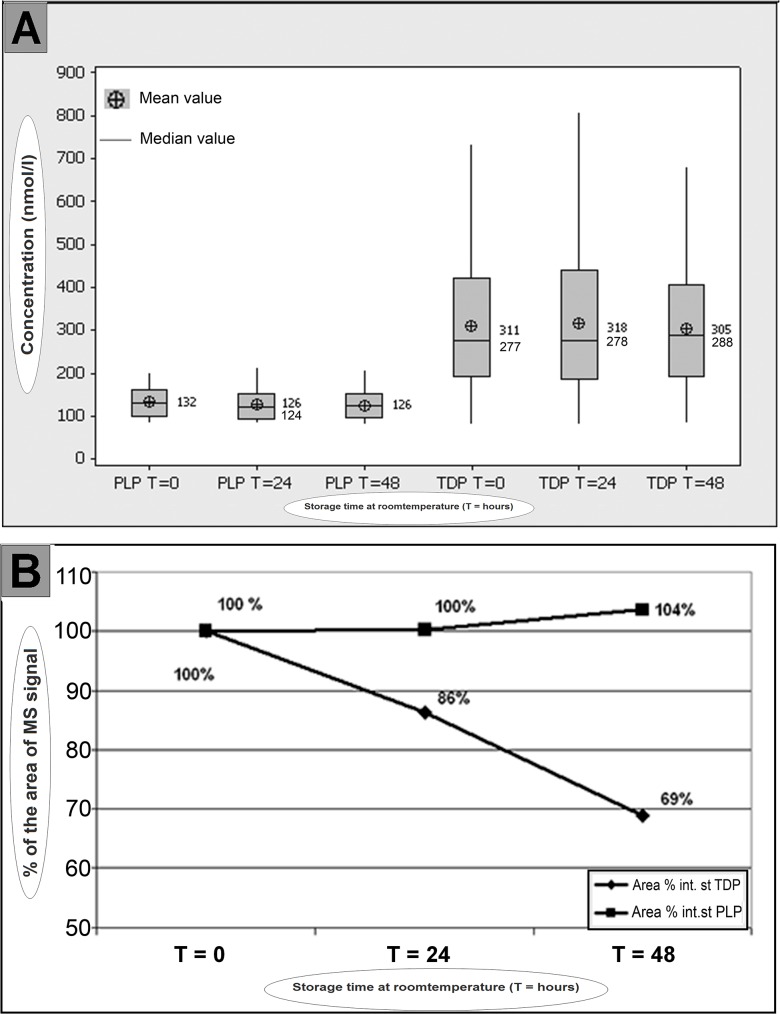
Stability of PLP and TDP estimated in processed blood samples (A) and their internal standards (B) which were stored for 24 and 48 hours at room temperature in the dark. **A: in processed blood samples.** Box is presenting the 25 ^th^ and 75 ^th^ percentile (P25 and P75). The length of the box is the interquartile range of the concentration and contains 50 percent of the cases. The lines protruding from the box go out to the smallest and largest concentrations. **B: internal standards of TDP and PLP.**

Thiamine is one of the least stable water-soluble vitamins at neutral matrix pH [7]. Maximum stability is obtained between pH 2.0 and 4.0. To assess the stability of PLP and TDP, a total of 96 samples where analyzed after 24 hours and 48 hours storage at room temperature in the auto-sampler in the dark. Fig 4 shows that the PLP and TDP concentration can be measured adequately over a period of 48 hours, when corrected by an internal standard.

## Discussion

To accommodate the high number of vitamin B1 and vitamin B6 analyses in our laboratory there is an urgent need for a new method with short chromatography and turn around time. We developed an accurate, rapid and sensitive UHPLC-MS/MS method, for simultaneous quantification of TDP and PLP in human whole blood. Combined analysis of TDP and PLP in whole blood by UHPLC-MS/MS has not been described before. Our method used whole blood rather than washed erythrocytes to reduce pre-analytical hands on time. It did not require derivatization and we used a simple mobile phase without the ion pairing reagent HFBA. The use of ion-pairing reagent should be avoided because of contamination of the mass detector. We show that TDP and PLP can now be quantified in a single run, which has superior throughput compared to HPLC.

The disadvantage of the current HPLC methods for measuring TDP and PLP is the need for pre- or post column derivatization. Sample preparation may introduce human errors which are not noticed due to lack of proper internal standards in the HPLC methods. Moreover, sample preparation is time consuming [11–25].

Since LC-MS/MS is an excellent method to quantitatively estimate different components in the same run without the need for derivatization and sample preparation, our goal was to develop an UHPLC-MS/MS based method for establishing vitamin B1 and B6 status in patients. In literature several LC-MS/MS methods have been described for measurement of PLP [28], vitamin B6 vitamers in plasma [29–30], liquor [31] or water-soluble vitamins in selected food matrices [32].

Midttun et al [29] developed a LC-MS/MS method for analysis of vitamin B2 and B6 not only in the active form but also in the inactive forms in human plasma. Gentili et al [32] showed that water-soluble B vitamins, including B1 (thiamine) and B6 (5 vitamers), can be simultaneously estimated in food matrices by detection with a photodiode array in sequence with a triple-quad MS-Multiple Reaction Monitoring.

Whether all B6 vitamers or only plasma PLP is the best predictor of B6 status has not been elucidated yet [33]. It has been suggested by Bates [34] and Leklem [35] that plasma PLP is sufficient for clinical practice. In plasma, vitamin B6 is present only as PLP and PL [29], of which PLP is the catalytically active form of vitamin B6 [8]. TDP is the main form present in whole blood and it reflects the concentration in erytrocytes [12]. Therefore, we used whole blood to estimate vitamin B1 (TDP) and B6 (PLP) status. The measurement of the inactive B vitamins is only desirable in rare metabolic enzyme deficiencies. Hence, only the active forms vitamin B1 and vitamin B6 i.e. TDP and PLP are of interest to establish the nutritional status of patients.

In the newly developed method we selected internal standards (PLP-D_3_ and TDP-D_3_) with similar behaviour as the target analytes (PLP and TDP) in sample preparation, injection and analysis in the UHPLC-MS/MS to allow correction of analytical variation. Furthermore, the use of internal standards adjusts for ion suppression when present in the same matrix as the analyzing target analytes (PLP and TPD). The internal standards (PLP-D_3_ and TDP-D_3_) are stable on storage for several month, which is an advantage. In contrast, our current HPLC semicarbazide and thiochrome method lacks an internal standard, because adequate internal standards are not available. For thiamine analysis, salicylamide, sodium salicylate, and anthracene have been used for injection volume correction as these compounds fluoresce similarly to thiochrome [36]. For PLP analysis, 4-deoxypyridine has been added, but the amount required to provide sufficient fluorescence response can interfere with the elution of low levels of metabolites in some systems [37].

We performed simple protein precipitation of the sample only. Although protein precipitation does not remove lipids, this was sufficient to provide good sample quality. Moreover, lipid removing plates are based on the use of methanol in higher concentrations (3:1 MeOH) than our UHPLC-MS/MS method (2%) concentration, which is inconvenient.

The imprecision of the new method was within set limits i.e. <7.7% for TDP and <10.4% for PLP and was better compared to the HPLC method for TDP [11] and PLP [38]. The method is linear far above the physiologically relevant concentrations: up to 1153 nmol/L for TDP and up to 2864 nmol/L for PLP. Patient samples analyzed by the HPLC thiochrome and semicarbazide method and by the UHPLC-MS/MS method showed comparable results. The samples of our national proficiency testing program for TDP also showed identical results for UHPLC-MS/MS and the consensus method (HPLC). The agreement between methods was not as good for PLP in the samples of our national proficiency testing program, which was due to two samples with the very low concentrations. These samples showed a much higher concentration estimated with the UHPLC-MS/MS assay.

Excluding these samples revealed a slope of 0.89 with an intercept of 8.00 for PLP. Close investigation of the two samples of our national proficiency testing program with the lowest concentrations revealed much higher internal standard areas as compared to the areas in blood samples and the other samples of our national proficiency testing program. This suggests that the matrix in the two samples of our national proficiency testing program with low concentrations of PLP was different. Performing a standard addition curve for PLP we found a slope of 1.06 and intercept of -2.00 (Fig 3). Hence, samples other than blood (with a different matrix) are not suitable for our method, but can still be measured by standard addition. We observed the same phenomenon in commercially available control samples (data not shown). The performance of quality control samples does not address accuracy unless these samples are comparable to blood matrix samples [39].

Interference was not investigated since vitamins (B2, B3, and B5) and common medication such as caffeine, acetaminophen, aspirin, diphenhydramine, ephedrine, ibuprofen or naproxen have different *m/z*.

In conclusion, we developed a highly sensitive and specific UHPLC-MS/MS method suitable for simultaneous analysis of TDP and PLP in human whole blood.

In the new UHPLC-MS/MS method we used simple sample preparation, no derivatization of TDP and PLP and added internal standards to correct for sample handling. The method can be easily implemented in routine diagnostic settings. LOQ of TDP and PLP were 9.4 nmol/L and 25.9 nmol/L, respectively. The CVs of TDP were <7.7% and of PLP <10.4%. With a 2.5 min run time, the method is an excellent alternative for the currently used HPLC methods. The present study showed comparable results between UHPLC-MS/MS and HPLC in patient samples, but identified some performance differences of the samples of our national proficiency testing program and commercial available control levels. Matrix differences, which can lead to inaccurate results, must be recognized.
